# Bilateral adrenal hemorrhage in the background of *Escherichia coli* sepsis: a case report

**DOI:** 10.1186/s13256-017-1236-0

**Published:** 2017-03-17

**Authors:** Jahanzaib Khwaja

**Affiliations:** St Helier Hospital, Wrythe Lane, Carshalton, SM5 1AA UK

**Keywords:** Bilateral adrenal hemorrhage, Sepsis, Antiphospholipid syndrome, Anticoagulation, Thrombosis

## Abstract

**Background:**

Sepsis is a syndrome of life-threatening organ dysfunction caused by a dysregulated host response to infection. It can have devastating consequences, including bilateral adrenal hemorrhage, particularly in patients at high thrombotic risk, such as those with antiphospholipid syndrome and those on long-term anticoagulation.

**Case presentation:**

A 49-year-old white woman re-presented to hospital with a history suggestive of sepsis. She had a medical background of primary antiphospholipid syndrome on lifelong warfarin. Ten days prior to this presentation, she had been hospitalized following *Escherichia coli* bacteremia, commenced on intravenous antibiotics, and discharged 2 days later with a prescribed 5-day course of oral amoxicillin. On readmission, she had ongoing fever, myalgia, malaise, and hypotension. Investigations revealed anemia with thrombocytopenia, hyponatremia, and acute-on-chronic kidney injury. Despite treatment for urosepsis, she became tachypneic, clammy, light-headed, drowsy, and hypothermic. Computed tomography revealed bilateral adrenal hemorrhage, and biochemical examination confirmed hypoadrenalism. Following discharge, she had persistent renal and hepatic injury lasting 3 months.

**Conclusions:**

Early identification, intensive monitoring, and aggressive support may reduce the acquired thrombotic risk and avoid potentially life-threatening outcomes of sepsis.

## Background

Sepsis is a syndrome of life-threatening organ dysfunction caused by a dysregulated host response to infection [1]. It carries significant hospital mortality and can have devastating consequences, particularly in patients at high thrombotic risk, such as those with antiphospholipid syndrome (APS). Adrenal hemorrhages are rare, occurring in 0.4% of patients with APS, and little literature details precipitating factors [2]. I report one such case and the role of sepsis in inducing it.

## Case presentation

A 49-year-old white woman re-presented to hospital with a history suggestive of sepsis. She had a background medical history of primary APS on lifelong warfarin for over 20 years with good compliance, hypertension treated with ramipril, chronic kidney disease, and a bicuspid aortic valve causing moderate regurgitation. She exercised regularly, was a nonsmoker and drank alcohol rarely. She was on neither hormone replacement therapy nor oral contraceptives.

Ten days prior to this admission, she had been hospitalized following a 1-day history of high-grade fever, nausea, urinary frequency, back pain, and rigors that had commenced 2 days after returning from a vacation in Mexico. She was not exposed to freshwater or jungles. She was hemodynamically stable and alert. Her urine culture was positive for coliform species, and blood cultures revealed an *Escherichia coli* bacteremia. The result of an abdominal ultrasound was normal, and transthoracic echocardiography visualized no vegetations and normal left ventricular dimensions with preserved systolic function. She was commenced on intravenous piperacillin-tazobactam and fluids and improved promptly, enabling discharge 2 days later with a prescribed 5-day course of oral amoxicillin.

**Fig. 1:**
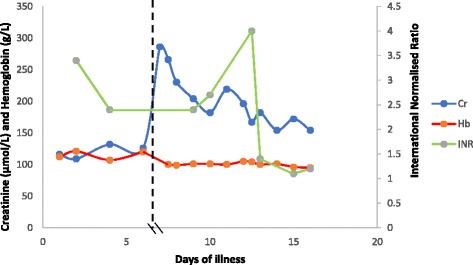
Timeline of the patient’s clinical course. *Cr* Creatinine, *Hb* Hemoglobin, *INR* International normalized ratio

Ten days later she self-presented to hospital with ongoing fever, myalgia, and malaise. Her urinary frequency had stopped; however, she complained of intermittent abdominal pain and episodes of vomiting and diarrhea. A medical practitioner had reviewed her case a few days prior and had diagnosed gastroenteritis. On examination, she was hypotensive (105/52 mmHg) with a normal heart rate (85 beats per minute), her respiratory rate was 22 breaths per minute, she had 98% oxyhemoglobin saturation on room air, and she was afebrile (37.3 °C). Her abdomen was soft and nontender without organomegaly. Intravenous fluids and antiemetics were instituted to good effect. No skin or buccal pigmentary changes or stigmata of infective endocarditis were noted. Investigations showed she had microcytic anemia, mild thrombocytopenia, hyponatremia, and acute-on-chronic kidney injury. The results of the patient’s blood tests done at admission are shown in Table 1, and her clinical course is shown in Fig. 1. Urinalysis showed 3+ blood, 2+ protein, and 2+ leukocytes, with no ketonuria or glycosuria by dipstick. The result of a pregnancy test was negative. Her electrocardiogram and chest x-ray were unremarkable.

**Table 1:** Blood laboratory examination results

Variable	Reference range	First admission	Readmission
Hemoglobin, g/L	115–145	112	100
Hematocrit, L/L	0.36–0.46	0.33	0.29
Mean cell volume, fL	84–98	82	79
White cell count, ×10^9^/L	3.5–10	11.2	4.5
Differential count, %			
Neutrophils, ×10^9^/L	1.7–7.5	9.2 (82)	2.4 (53)
Lymphocytes, ×10^9^/L	1.0–3.5	1.0 (8.9)	1.3 (28)
Monocytes, ×10^9^/L	0.3–1.0	1.1 (9.8)	0.6 (13)
Eosinophils, ×10^9^/L	<0.4	0.0 (0.0)	0.2 (4.4)
Basophils, ×10^9^/L	<0.1	0.0 (0.0)	0.0 (0.0)
Platelet count, ×10^9^/L	150–400	166	147
C-reactive protein, mg/dl	0–5	151	76
Sodium, mmol/L	135–145	135	130
Potassium, mmol/L	3.5–5.0	4.0	4.5
Urea, mmol/L	1.7–6.8	10	16.2
Creatinine, μmol/L	Baseline 110	116	286
Total bilirubin, μmol/L	0–20	19	9
Alanine aminotransferase, U/L	10–35	160	73
Alkaline phosphatase, U/L	35–104	111	419
Albumin, g/L	34–50	34	28
Erythrocyte sedimentation rate, mm/hour	1–20	75	78
International normalized ratio	Target 2.5–3.5	3.4	2.4

The patient was admitted to the acute medical ward. Two blood cultures and one urine culture were negative. Test results for other infectious pathogens (malaria, rickettsia, dengue, West Nile virus, chikungunya, Eastern equine encephalitis virus, yellow fever) were negative. An abdominal ultrasound scan displayed an oval 20 × 40-mm echo poor area above the right kidney, with some debris within it. This had not been present on the previous ultrasound 2 weeks prior.

**Fig. 2:**
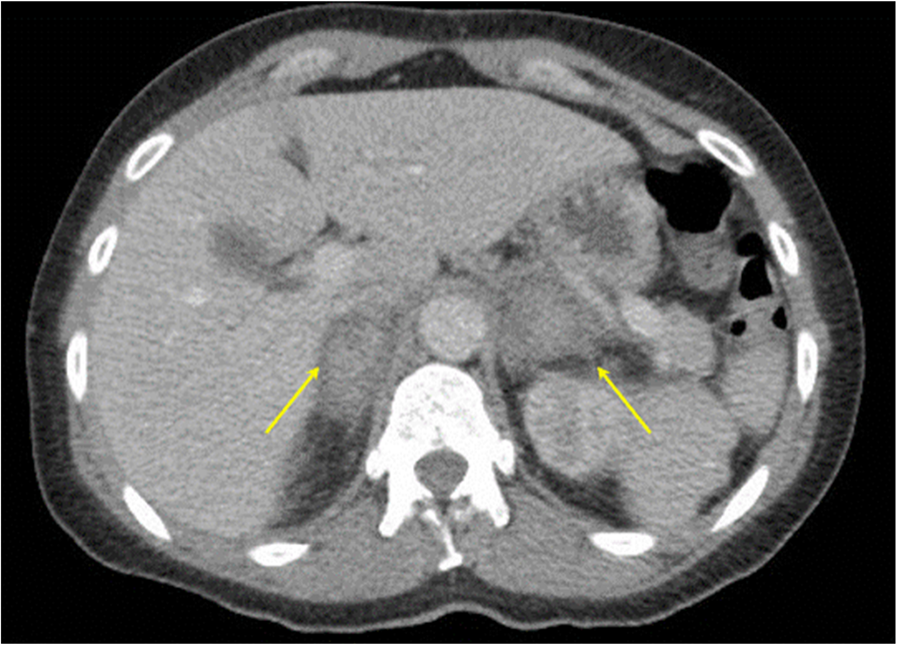
Computed tomography of the abdomen and pelvis with intravenous contrast showing bilateral adrenal hemorrhages (*arrows*)

The patient was prescribed intravenous piperacillin-tazobactam for presumed urosepsis. However, on the second day of treatment, she developed chills, shivering, and tachypnea (respiratory rate 25 breaths per minute), yet maintained her blood pressure (160/80 mmHg). She was clammy, light-headed, drowsy (Glasgow Coma Scale score 14/15), and hypothermic (33.3 °C). Her capillary blood glucose level was 6.2 mmol/L. An urgent dose of intravenous gentamicin was given. An urgent computed tomographic scan of the patient’s abdomen and pelvis revealed bilateral adrenal enlargement with high density centrally consistent with hemorrhage (Fig. 2). Blood test results confirmed primary hypoadrenalism with low early morning cortisol (33 nmol/L), high adrenocorticotropic hormone (943 ng/L; normal range 10–50 ng/L), and negative adrenal antibodies with normal thyroid function, luteinizing hormone, and follicle-stimulating hormone levels. Her hemoglobin level was 100 g/L, her platelets were 218 × 10^9^/L, her international normalized ratio was 4.0, and her fibrinogen level was 5.8 g/L (normal range 1.8–4.5 g/L).

The patient was treated with intravenous hydrocortisone 100 mg three times daily, followed by oral therapy. This resulted in prompt improvement of her symptoms. Warfarin was withheld, and 1 mg intravenous vitamin K reversal was given. Prophylactic low-molecular-weight heparin was later initiated, and follow-up in the thrombosis clinic included reloading on warfarin.

Three months later a repeat computed tomographic scan showed no further bleeding and some reduction in size of the adrenal glands. The acute-on-chronic deterioration in renal function gradually stabilized at a lower glomerular filtration rate. A renal biopsy showed some scanty mesangial deposits. No reversible cause of renal impairment and no evidence of underlying connective tissue disease or overt thrombosis were identified. Elevated transaminases progressed (alanine aminotransferase peaked at 846 U/L) and sequentially normalized without medical intervention. An acute liver screen including ultrasound of portal and hepatic veins was unremarkable. Six months later the patient was well and continued to have follow-up with the hematology, endocrinology, and renal physicians.

## Discussion

This report describes a patient with bilateral adrenal hemorrhage following a recent admission with *Escherichia coli* sepsis. The background of APS and anticoagulation put this patient at high risk of developing serious complications of sepsis. These critical factors are discussed further.

The Third International Consensus Definitions for Sepsis and Septic Shock Task Force recently clinically characterized sepsis by a rise in Sepsis-related Organ Failure Assessment score ≥2 [1]. Although our patient was initially stable, she developed new evidence of organ dysfunction, namely hypotension, increased work of breathing, thrombocytopenia, and renal impairment. This differentiates uncomplicated infection from sepsis, which represents a ≥10% risk of in-patient mortality. Moreover, following discharge, the patient developed significant subsequent renal and hepatic impairment persisting over 3 months. In view of this, during initial admission, a longer duration of intensive monitoring, aggressive support, and longer duration of antibiotics are strongly advocated in these patients.

Because sepsis is associated with activation of procoagulant and fibrinolytic pathways and a downregulation of anticoagulant pathways, it represents a risk factor for thrombosis. Classically, infection-driven adrenal hemorrhage is associated with meningococcal septicemia, the so-called Waterhouse-Friderichsen syndrome. However, an adrenal histopathology series of 65 patients with fatal bacterial infections showed a broad collection of pathogenic bacteria (both gram-positive and gram-negative), including *Streptococcus pneumoniae*, *Staphylococcus aureus*, *Klebsiella* spp., and *Legionella* spp., the majority resulting in adrenal hemorrhage [3]. Our patient presented 14 days prior to diagnosis of adrenal hemorrhage with infection caused by *Escherichia coli*. Lipopolysaccharide is a ubiquitous constituent of the cell wall of gram-negative bacteria that can bind to endothelial and inflammatory cells as a highly potent activator, leading to increased transcription of proinflammatory cytokines and other mediators.

The patient had additional risk factors for adrenal hemorrhage related to both APS and the long-term use of anticoagulation with warfarin. Adrenal hemorrhages have been described in nonseptic hypercoagulable states, including heparin-induced thrombocytopenia [4], and in a patient with factor V Leiden, protein C resistance, and elevated factor VIII [5].

APS is a systemic disorder with varied clinical features. Our patient was diagnosed over 20 years ago, having had two first-trimester miscarriages and a second-trimester fetal loss, retinal vein occlusion, livedo reticularis on her upper arms and thighs, migraines, labile hypertension, aortic incompetence, and chronic kidney disease. APS is the commonest cause of acquired thrombophilia and is antibody-mediated. This occurs in the presence of autoantibodies (lupus anticoagulant, anticardiolipin, and anti-β_2_-glycoprotein I) against plasma proteins with an affinity for phospholipids, such as β_2_-glycoprotein I [6]. Although the pathogenesis is not fully understood, it is hypothesized that by upregulating tissue factor and thromboxane synthesis in endothelial cells, monocytes, and platelets, these antibodies cause a thrombogenic state. Of note, in a rat model, infusion of antiphospholipid antibodies alone did not cause thrombosis, but this was initiated only after injection of lipopolysaccharide [7]. Lipopolysaccharide may cause conformational change in β_2_-glycoprotein I from closed to open, so binding sites to autoantibodies are exposed and prothrombotic pathways are activated [8].

Thrombosis in APS most frequently affects the deep venous system but can occur in any vessel of any size [9]. Adrenal hemorrhage in this case may be a consequence of adrenal vein thrombosis. The absence of pigmentary buccal or skin changes caused by adrenocorticotropic hormone excess indicates that adrenal involvement was acute. A growing number of case reports in the literature demonstrate acute bilateral adrenal hemorrhage in APS [10–13]. The adrenal glands have a limited venous drainage with an eccentric muscular arrangement. Accordingly, they are susceptible to turbulent flow or stasis and thus to platelet thrombi within the adrenal vein [14]. Hemorrhage may be a secondary event [15]. Alternatively, spontaneous hemorrhage without preceding thrombosis has also been described [16].

Additional factors in this patient included anticoagulant therapy and coexistent thrombocytopenia, which is found in 20% of patients [9]. This, however, is usually seen in APS secondary to other autoimmune conditions, such as systemic lupus erythematosus, which was not present in this patient.

## Conclusions

Bilateral adrenal hemorrhage is a rare cause of primary adrenal insufficiency. The risk is likely to be heightened in patients at increased risk of thrombophilia, such as those with APS, especially when a severe infection intervenes. This case highlights the serious secondary consequences of sepsis, which should be considered in high-risk patients. Early identification, monitoring, and management of sepsis may reduce this acquired thrombotic risk and avoid potentially life-threatening outcomes.

## Abbreviations

APS: Antiphospholipid syndrome
INR: International normalized ratio
